# Impaired Expression of Cytokines as a Result of Viral Infections with an Emphasis on Small Ruminant Lentivirus Infection in Goats

**DOI:** 10.3390/v8070186

**Published:** 2016-07-05

**Authors:** Justyna Jarczak, Jarosław Kaba, Daria Reczyńska, Emilia Bagnicka

**Affiliations:** 1 Institute of Genetics and Animal Breeding, Polish Academy of Sciences, Jastrzebiec, ul. Postepu 36A, 05-552 Magdalenka, Poland; j.jarczak@ighz.pl (J.J.); d.reczynska@ighz.pl (D.R.); 2 Laboratory of Veterinary Epidemiology and Economics, Faculty of Veterinary Medicine, Warsaw University of Life Sciences, ul. Nowoursynowska 166, 02-787 Warszawa, Poland; jaroslaw_kaba@sggw.pl

**Keywords:** goat, SRLV, gene expression, cytokine

## Abstract

Knowing about the genes involved in immunity, and being able to identify the factors influencing their expressions, helps in gaining awareness of the immune processes. The qPCR method is a useful gene expression analysis tool, but studies on immune system genes are still limited, especially on the caprine immune system. Caprine arthritis encephalitis, a disease caused by small ruminant lentivirus (SRLV), causes economic losses in goat breeding, and there is no therapy against SRLV. The results of studies on vaccines against other viruses are promising. Moreover, the Marker-Assisted Selection strategy against SRLV is possible, as has been shown in sheep breeding. However, there are still many gaps in our knowledge on the caprine immune response to infection. All types of cytokines play pivotal roles in immunity, and SRLV infection influences the expression of many cytokines in different types of cells. This information encouraged the authors to examine the results of studies conducted on SRLV and other viral infections, with an emphasis on the expression of cytokine genes. This review attempts to summarize the results of studies on the expression of cytokines in the context of the SRLV infection.

## 1. Introduction

Small ruminant lentivirus (SRLV) causes chronic diseases in goats (caprine arthritis encephalitis, CAE), sheep (maedi-visna disease, MV) and other small ruminants, including wild animals [1]. There is no specific therapy for SRLV-infected animals, and there is no vaccine available to prevent infection [1,2]. Studies on preventing and eradicating SRLV are becoming increasingly urgent, especially since the discussion, although still controversial, on the possibility that genomic evolution of these viruses, similar to that which occurred in HIV, has already begun (there is a fear that the goat/sheep-human interspecies barrier will be crossed). Nevertheless, the rapid evolution of retroviruses in the host (together with latency, persistence, and the inability of immune systems to combat these types of viruses) is their main feature [1]. Because food safety is a priority in developed societies, a knowledge of the immunological processes associated with SRLV infection is of special importance.

New methods of molecular genetics such as genomics, transcriptomics and proteomics allow for the exploration of the biology of viruses and their life cycles, and provide answers to the questions of how an infected organism copes with an infection, and which elements of the immune system respond to a viral infection. Is there any chance of a cure, and what could it be? Taking into consideration human diseases such as acquired immunodeficiency syndrome caused by HIV, cervical cancer (developed by human papilloma virus), and hepatitis type C (hepatitis C virus infection), these questions are essential. However, they are also important in the case of animals because of the huge costs caused by viral diseases. SRLVs cause chronic disease processes in goats, sheep and other small ruminants, including wild animals [1]. At present, there is no specific therapy for SRLV-infected animals, and there is no vaccine available to prevent infection. Nor is there any treatment to at least lower the viral load in the host. There may, however, be a chance to help protect animals from suffering, emaciation and reduction of production by lowering the viral load, because as Ravazzolo et al. [3] pointed out, goats with clinical signs of arthritis have a higher viral load and higher antibody titers than asymptomatic infected animals. Marker-assisted selection (MAS) offers another possibility to breed healthy animals, since it was found to use the polymorphism of the transmembrane protein 154 (*TMEM154)* gene as a marker for lentivirus-resistant sheep, followed by cases of scrapie in selective breeding [4]. Moreover, the study on cytokine gene and cytokine receptor gene polymorphisms in sheep indicates an association between gene variants and the level of cytokine gene transcripts [5,6]. The differences between breeds of sheep in resistance to SRLV is well known, and the polymorphism of transmembrane protein genes such as *TLR-7, -8* (toll-like receptors 7 and 8), *CCR5* (C-C chemokine receptor type 5), *MHC* (major histocompatibility complex), *TMEM154* and *38A* (transmembrane proteins: 154 and 38A), were shown to be associated with resistance to the lentiviruses [6,7,8,9,10]. Common diseases occurring in goat and sheep herds caused by lentiviruses lead to economic losses for breeders [4,11] and create a need for a better understanding of the epidemiology, course of infection, and host cell responses to infection.

Despite several studies on SRLV biology and its influence on the host, the immune response to virus infection is still not fully understood. Some information can be obtained from studies on HIV, due to the large similarity between HIV and SRLV. However, these are two distinct viruses and there are many differences in their biology and functioning. The study of immune processes at the molecular level has become extremely urgent nowadays. Knowing about the genes involved in immunity, and being able to identify factors that influence them and their protein product expressions help in gaining awareness of the immune processes in organisms. The real-time PCR method, also called qPCR, is a very useful tool for analyzing gene expressions, but the analyzing of immune system gene expressions is still limited, especially in the caprine immune system, including those analyses connected with SRLV infection.

SRLV infection influences the expression of many cytokines in different types of cells belonging to the immune system of the host. On the one hand, all these cytokines play a pivotal role in the immune system, including the responses to different viral infections. On the other hand, the cytokine genes are considered potential adjuvants in experiments on DNA vaccines against SRLV [2].

All this information encouraged the authors to examine the results of studies conducted on SRLV and other viral infections, with a special emphasis on the expression levels of cytokine genes.

This review attempts to summarize the results of studies on gene expression, especially cytokines, in the context of the small ruminant lentivirus infection.

## 2. Cytokines

Cytokines are small (most of them less than 30 kD) soluble proteins, or glycoproteins, released by various types of cells. They act as specific messengers that enable interactions between cells in both physiological and pathological conditions. Through specific cellular receptors, they regulate the intensity and duration of every stage of the immune response, particularly by modulating the balance between humoral and cell-mediated responses [12,13].

The innate response is the first line of defense. It is activated early to suppress the invasion and replication of invasive agents, including viruses. The more specific protection of the adaptive immune system is activated later [14].

Well over 100 cytokines have so far been discovered and their number is constantly increasing. Because of their prominent role in immune processes they are often called “hormones of the immune system” or “hormone-like proteins” [12,13]. They are divided into various groups on the basis of their source, sequence homology, receptor chain similarities, and functional properties. The following groups are commonly accepted: interferons (IFN-α, -β, -γ, -δ, -ε, -ω, -κ, -T and -ζ; for detailed information see Genin et al. [15]), interleukins (IL- about 50 interleukins altogether; for details see Akdis et al. [16]), chemokines with their four subfamilies (CXC, CC, XC, CX3C—approx. 50 altogether), the tumor necrosis factors superfamily, with members such as alpha (TNF-α) and beta (TNF-β), and others, such as the transforming growth factor beta (TGF-β), macrophage migration inhibitory factor (MIF) and hematopoietic growth factors [12,13,17].

Interferons are produced by different types of cells. IFN-α, -γ and IFN-β are responsible for the response to viral and bacterial pathogens and parasites. Interferons perform multiple functions during viral infections, e.g., inhibiting viral replication and spread in an organism. Furthermore, they modulate immunity, both innate mechanisms and adaptive response, shifting it towards cellular cytotoxity, and exert an anti-proliferative influence on some kinds of cells [18]. They are also involved in regulating restriction factor expression thereby inducing apoptosis of infected cells and cellular resistance to viral infection [19]. Moreover, they are able to interfere with virus replication, which is important in inducing anti-viral states in nearby uninfected cells [20]. A wide range of RNA and DNA viruses are sensitive to IFN-αand IFN-β-mediated anti-viral effects [21]. Another very important anti-viral function is natural killer (NK) cell stimulation. Thus, their activity against sensitive target cells is increased and they can lyse a wide range of cells, including virus-infected ones. This innate response is activated endogenously, in the early stages of viral infection [20]. Interferons are used to treat viral infections, some types of neoplasms, and multiple sclerosis [18].

Interleukins are numbered from 1 to 38. There are, however, 48 interleukins. That is because the names interleukin 1 (IL-1) and interleukin 17 (IL-17) cover two families of cytokines. Their main functions involve regulating cell growth, differentiation, motility, activation, releasing signaling proteins, and expressing receptors. This family of proteins comprises the main regulators of the immune and inflammatory responses acting on all involved cells. Some participate in stimulating inflammation (pro-inflammatory cytokines, e.g., IL-1α, IL-1β, IL-2, -6, -8, -12, -17, -23). The others inhibit the acute inflammatory response (anti-inflammatory cytokines e.g., IL-4, -10, -13). Each interleukin influences a very specific group of cells (with an appropriate receptor). Detailed information on the functions of interleukins is summarized by Lilesand van Voorhis [22], and complemented by the latest discoveries by Clavel et al. [23].

In addition to approx. 50 chemokines, 20 of their receptors have so far been identified in humans. Chemokines have a relatively small molecular weight (approx. 8–14 kDa; 70–130 amino acids) and were initially called “small cytokines.” Their most salient feature is the presence of four cysteines forming two sulphide bridges. They are mainly released by monocytes and macrophages, but almost any cell, given proper stimulation, can be a source of chemokines. Inflammatory (induced) and homeostatic (constitutive) expressions of chemokines can be distinguished. They act chemotactically, but sometimes activate different types of leukocytes, including monocytes, lymphocytes, neutrophils, and eosinophils to mediate inflammation [13,24]. Chemokines were originally described as mediators of immediate immune cell migration to inflammation or injury sites. Their role is connected with wound repair, pathogen entry, tumor metastasis, allergies, cardiovascular diseases, embryogenesis, organ development, angiogenesis, and inflammation. They can identify the site of injury by inflammatory cells and engage the specific leukocyte subpopulations. This is the first step to removing the attacking pathogens, eradicating injured cells and tissues, and repairing wounds [22] (http://www.uniprot.org/uniprot/).

## 3. Impact of Viral Infections on Expression of Cytokine Genes

Viral infections can induce various cytokine responses. This is probably because disparate viruses have distinct chemical structures and antigens, and can infect diverse host organs and cells [20]. The expression of pro-inflammatory cytokines and the balance between them and regulatory cytokines are essential in raising the innate and adaptive immune response [25]. Macrophages and monocytes are the main producers of cytokines involved in early intracellular antiviral response [20] such as the mechanisms involved in altering MHC expression, adhesion and co-stimulatory molecules, and directly activating or deactivating immune cells. The effects of these processes can lead to the activation of cellular antiviral responses involving NK cells, CTL (cytotoxic T lymphocyte), and antibody-mediated virus clearance [26].

The influence of viral infection on gene expression(mainly cytokines) has been studied, inter alia, in mice and humans [27,28], pigs [25], and sheep and goats [29,30,31].

One of the pro-inflammatory cytokines in humans, viz. interleukin-32 (IL-32), has been described as important in the response against *Mycobacterium tuberculosis,* H1N1 influenza A virus and vesicular stomatitis virus (VSV) [32]. IL-32 level increases during H1N1 influenza A infection, This correlates with IL-6 expression, which suggests that IL-6 induction is affected by IL-32. The production of IFN-α, IL-6 and TNF-α have been stimulated to trigger an anti-viral response during SRLV infection, via toll-like receptors 7 and 8 [33]. Several studies on the influence of HIV infection on cytokine expression in humans have shown that virus infection induces the expression of immunosuppressive cytokines such as IL-10, and pro-inflammatory ones, such as IL-1β, in monocytes or IL-6 in astrocytes [28,34]. Elevated TNF-α levels have also been observed in infected patients. However, Nath et al. [34] demonstrated increased IL-1β mRNA expression but a lack of this protein production in astrocytic cells. There are also some indications suggesting the inhibitory activity of IFN-α and IL-27 in the response against HIV [35]. Chemokines play an important role in the immune response of the host during infection, including those caused by HIV-1, respiratory syncytial virus (RSV), and cytomegalovirus (CMV). The C-X-C chemokines, together with IL-8, increase cytomegalovirus load and promote its dissemination in the body [36], while C-X-C and C-C chemokines are involved in inducing changes that result in the clinical manifestation of inflammation [27]. The level of transcripts of some chemokines from the C-X-C, C-C and C families could serve as a marker of RSV load and lung inflammation intensity in humans and mice [27,36]. Some chemokines (MIP-1α, MIP-1β, RANTES) act as HIV-1 suppressive factors [37]. Others, however, facilitate HIV-1 cell infection. This is because some chemokine receptors, e.g., CCR2, CCR3, CCR5 and CXCR4, support fusion between the cell and the virus [38]. Elevated level of chemokines, especially those from the C-C family, but also from the C-X-C (MIP-1α, MIP-1β, RANTES, MCP-1, MCP-3, MIP-3α, IP-10) families, and even some others, e.g., IL-8, are produced by human macrophages infected with influenza A or Sendai viruses [39]. These results were confirmed in a study by Julkunen et al. [40]. It proved that some chemokines (RANTES, MIP-1α, MCP-1, MCP-3, and IP-10), as well as other cytokines (IL-1β, IL-6, IL-18,TNF-α, IFN-α, IFN-β), are produced in infected cells. Many studies on cytokine expression in infected humans have been summarized in several works, e.g., Parienti [41], Connolly et al. [42] and Vandergeeten et al. [44].

Patel et al. [30] studied the cytokine expression profile (IL-4 and IFN-γ) during peste des petits ruminants (PPR), a viral disease of small ruminants caused by the PPR virus, a member of the *Morbillivirus* genus. It mainly occurs in Africa and the Middle East, but has also been found in India. The major symptoms are necrotizing, erosive stomatitis, enteritis and pneumonia. The correlation between the cytokine profile and the kinetics of the antigen and antibody of PPR-infected and vaccinated goats indicates that IFN-γ expression is more apparent in the infected group than the vaccinated one during the early phases of the disease. However, even within the infected group, a slight up-regulation in the early phase, and no expression in the intermediate and late phases of infection were observed. IL-4 expression was up-regulated in the early phase and down-regulated in the late phase of the disease, but the results of the intermediate phase are more pronounced in the infected group than in the vaccinated group. The ability to stimulate a functional Th2 response (type 2T helper cells that produce cytokines, elicit predominantly humoral immunity, e.g., IL-4) after two weeks from the beginning of infection may be an essential factor in deciding the survival of PRR-infected animals. The study of Nfon et al. [29] on the Rift Valley fever virus (RVFV), a spherical enveloped and single-stranded RNA member of the *Bunyaviridae* family transmitted by mosquitos, also illustrates the impact of viral infection on the innate immune response. RVFV causes the spread of Rift Valley fever in humans and ruminants in parts of Sub-Saharan Africa (Kenya, Tanzania, and Somalia) and the Near-East (Yemen, Egypt, and Saudi Arabia). Innate and adaptive immune responses to the RVFV had previously been studied on animal models such as mice [44] and rhesus monkeys [45,46]. The studies on goats led to a comparison of the differences in the course of infection between animals infected by mosquitos and those infected with biological material e.g., blood serum derived from other animals IL-12 and IFN-γ levels peaked early after infection. The IL-12 peaked on the first day after infection in both infected groups, while the IFN-γ response was activated on the second (insect cell-derived RVFV) and fourth (mammalian cell-derived RVFV) days after infection. TNF-α, IL-6 and IL-1β activity was detected later, and showed a slight increase from the first to the sixth day after infection, with no differences between the groups being observed. The high serum IFN-γ level during RVFV infection in goats led to further in vitro research, although in this case IFN-γ acted minimally against RVFV.

SRLV infection activates both the innate and adaptive responses of the organism. Innate immunity activation is extremely important for stimulating the adaptive immune system. However, SRLVs weakly induce Type I IFN [47,48] (a major group of cytokines engaged in response against viruses [14]) from many infected cells, including macrophages, which are very sensitive to the influence of this type of interferon even as early as the monocyte stage. The almost complete inhibition of viral replication has been stated. This is probably connected with the interference of Type I IFN with the proliferation and differentiation of monocytes. Moreover, the lentivirus-induced interferon (LV-IFN), probably a mixture of IFN-γ I and II, is produced in SRLV-infected cultures in vitro. LV-IFN inhibits the proliferation of monocytes to macrophages and thus also inhibits virus replication in monocytes. LV-IFN also has a direct inhibiting influence on viral replication in mature macrophages [47,48]. Furthermore, SRLVs are sensitive to the activity of this kind of IFN in monocytes, and virus replication can be successfully reduced there.

Jarczak [49] found that SRLV infection causes decreasing expression of IL-1α, IL-1β and IL-6 on the transcript and protein levels, and no expression of IFN-γ and TNF-α on the protein level (despite the presence of their transcripts) in whole blood of animals. The transcript and protein levels of IFN-β were the same in infected and virus-free animals. The inhibition of many cytokine expressions in the blood may indicate the suppression of the immune response of the SRLV-infected organism. This observation is partly confirmed by the results obtained by Pyrah and Watt [50], who pointed out that SRLV-infected sheep have a lower immune response to mycobacterial antigens, which may be why the immunity of the host is affected.

Elevated expressions of TNF-α and Il-6, as well as IFN-γ, IL-1β, Il-4, IL-10, and IL-2 in lung tissues, were identified in microglia derived from VMV-infected sheep [51]. Lechner et al. [52] found that CAEV increases the constitutive expression of IL-8 and MCP-1 (monocyte chemoattractant protein 1) in macrophages infected in vitro, while it decreases the level of transforming growth factor β1 (TGF-β1), a member of the transforming growth factor beta superfamily of cytokines. SRLV-infected and uninfected macrophages showed different patterns of cytokine expression in response to infection with other pathogens. This may mean that the presence of lentiviruses in macrophages deregulates their functioning. This finding also confirms Jarczak’s [49] and Pyrah’s and Watt’s [50] observations. Moreover, an elevated IL-8 level seems to be a common attribute in lentivirus infections as HIV infection in humans and SRLV infection in sheep also increase the expression of this cytokine. Another study on cytokine expression in synovial membranes at different times after experimental intravenous and intracarpal infections revealed elevated levels of TNF-α, MCP-1 and IL-6 as early as six days after infection, and this increased expression persisted for over two years while the disease lasted. Lower levels of IFN-γ, IL-2 and IL-10 were sometimes stated as being due to no IL-8 expression. The expressions of these cytokines in lymph nodes were somewhat ambiguous [53].

Nimmanapalli et al. [54] studied the immunomodulatory effect of lentivirus infection on IL-16 (human recombinant IL-16, rhIL-16) expression in vitro, using cells derived from SRLV-infected and non-infected goats. The proviral DNA level was lower in caprine monocytes treated with rhIL-16. The increased IL-16 expression during the lentivirus infection might therefore have inhibited viral integration.

The preliminary studies of Ravazzolo et al. [3] on animals with different viral loads in their lymph nodes showed changes in the relative expressions of IFN-α, IL-12p40, IL-10, TGF-α. The expressions of those cytokines were increased two to 11-fold in the samples from the goat with the highest proviral and viral RNA loads. Nevertheless, IL04 expression also varied among the goats, although there was no correlation with viral load. However, interleukin-16 (IL-16), a pro-inflammatory cytokine produced by a variety of cells, including macrophages, showed increased expression (both mRNA and protein levels) in the blood of a SRLV-infected goat. These results are in agreement with the above-cited results obtained by Nimmanapalli et al. [54]. Higher IL-16 expression in SRLV-infected synovial membrane cells in vitro compared with uninfected control cells, was also stated by Sharmila et al. [55]. Moreover, increased IL-16 RNA levels were observed in the synovial fluid, serum, and culture supernatants of the PBMCs of infected animals, as compared to the samples from the control goats. The authors suggest that this cytokine is constitutively expressed at low concentrations in normal, uninfected PBMCs and synovial membrane cells. The higher IL-16 production during SRLV infection could be responsible for the increased lymphoid cell infiltrations observed in arthritic joints and other tissues.

The most recent results of the in vitro study on the influence of ovine progressive pneumonia virus (OPPV), which is also a member of the SRLV family, on gene expression in goat synovial membrane cells, indicate the important role of cytokines in the host response to the infection. Of the 22,000 genes studied, 657 were up-regulated and 889 down-regulated after 12 h post-infection. The response of the cells was reduced by 24 h and was stable until 48 h. This was probably because the immune response of the cells achieved a balance with the replication of the virus. Some of the most differentially expressed genes, e.g., CCL2, CCL5, CCL20, IL6, IL9, and IL16, belonged to the chemokine ligands or interleukins families [56].

There is no doubt that some cytokines can be produced as a response to virus-induced inflammation and can also help increase virus replication. GM-CSF (granulocyte macrophage colony-stimulating factor) and TNF-α [57], IL-1β [58], and IFN-γ [59], induce SRLV replication through virus gene promoter activation. Moreover, on the one hand, TNF-α stimulates the macrophages during the early response to infection [57], but on the other hand, macrophage activation, especially the maturation of blood monocytes to tissue macrophages, causes an increase in the replication of viruses within these cells [31]. Similar processes occur during HIV-1 infection, where the increase of virus replication in macrophages also requires cytokine and lymphocyte contact [60]. Thus, the cytokines protect the host against SRLV infection, but are simultaneously utilized by viruses to replicate their genomes [33].

Information on caprine cytokines is summarized in Table 1.

## 4. Conclusions

The information above reveals several mechanisms for activating pro-inflammatory, anti-viral cytokines and chemokines during viral infection. However, despite this extensive and intensive study, the information in the literature is still limited, and there remain many gaps in our knowledge on the gene expression profile in different virus infections. Further investigations are necessary to define the contribution of these mechanisms to the diseases associated with retrovirus infection. This is important for their possible future therapeutic use. Small ruminant lentiviruses (SRLVs) use the immune system cells, e.g., macrophages, to replicate themselves, and CD4+ T cells (without infecting them) to create the infection. Moreover, different types of infected cells act as viral reservoirs e.g., epithelial cells of the mammary gland [66], and the microglia cells and endothelial cells of the nervous system [67,68]. Peluso et al. [31] stated that the maedi-visna virus uses a “Trojan Horse” mechanism to spread itself in the host using monocytes. Viruses hidden inside the infected cells are undetectable by the immune system and cannot be combated [33]. This creates additional difficulties in fighting the disease. However, eradicating SRLV from goat and sheep herds has become increasingly important, especially as the issue of food safety is very important nowadays for health-conscious consumers, and the risk to humans from SRLV is being discussed. It is therefore crucial that every aspect of the lentivirus infection in the goat organism, especially the reaction of the immune system, be understood. Any solution to combat SRLV (vaccine or MAS) will require that the main genes involved in host defense against viral infection be identified, and that their expressions, both at the gene and protein levels, be analyzed in order to discover the interaction mechanisms between the host and pathogens. Potential differences could show how the inflammation changes and deregulates the response of the immune system of the host, and could help identify the genetic markers of viral resistance. The influence of SRLV infection on the gene and protein expressions involved in the immune system of the goat is a problem that has not been sufficiently studied. A vaccine against retroviruses, however, probably will still be difficult to invent. It would therefore seem that our current level of knowledge on introducing MAS to national breeding programmes of goat or sheep may provide tangible progress in eradication of this disease from herds, and that some cytokine genes could serve as candidate genes to be used in MAS to obtain animals with resistance to viral infection. Thus, their molecular characterization, including polymorphism and level of gene expressions on mRNA and protein levels, could help in developing new antiviral strategies in goat and sheep herds.

## Figures and Tables

**Table 1:** Functions of *Capra hircus* cytokines based on NCBI (http://www.ncbi.nlm.nih.gov/), UniProt (http://www.uniprot.org/uniprot/), and literature data.

Gene Name/Gene Symbol	GenBank/UniProt Accession	Main Functions	Known Connection with CAEV	Source
Interleukin 1 alpha/IL-1α	D63350/P79161	Produced by activated macrophages. Stimulates thymocyte proliferation by inducing IL-2 release. Stimulates B-cell maturation and proliferation. Stimulates fibroblast growth factor activity and release prostaglandin and collagenase from synovial cells.	Elevated expression in the blood of infected goats and the lungs of infected sheep	[49,51,61]
Interleukin 1 beta/IL-1β	D63351.1/P79162	As above.	Effect on VMV replication; elevated expression in the blood of infected goats on mRNA and protein levels, promote virus replication	[49,58,61]
Interleukin 2/IL-2	-/P36835	Immune response, growth factor, for lymphocytes, stimulates proliferation and differentiation of NK cells, used in treatment in cancer and AIDS	Not responsive to CAEV gp135 surface protein (SU) stimulation of PBMC derived from either asymptomatic or arthritic goats, elevated expression in the lungs of infected sheep.	[16,51,62,63]
Interleukin 4/IL-4	-/P79155	Participates in several B-cell activation processes. Co-stimulates DNA-synthesis. Induces the expression of class II MHC molecules on resting B-cells. Enhances the secretion and cell surface expression of IgE and IgG1. Regulates the expression of the low affinity Fc receptor for IgE (CD23) on lymphocytes and monocytes.	Varied expression between the goats, but does not correlate with viral load, increased expression in the lungs of infected sheep.	[3,51,64]
Interleukin 5/IL-5	-/B5LVN9	Immune response, acts on proliferation, activation, differentiation, survival and adhesion of eosinophils.	-	[16,64]
Interleukin 6/IL-6	D86569.1/Q28319	Potent induction of the acute phase response. Final differentiation of B-cells into Ig-secreting cells involved in lymphocyte and monocyte differentiation. Induces myeloma and plasmacytoma growth and nerve cell differentiation. Acts on B-cells, T-cells, hepatocytes, hematopoietic progenitor cells and cells of the CNS, as a myokine discharged into the bloodstream after muscle contraction, and increases the breakdown of fats and improves insulin resistance.	Elevated expression in the blood and synovial membranes of infected goats and in the microglia of infected sheep.	[49,51,52,61]
Interleukin 8/IL-8	-/-	Immune response, activates neutrophils, NK cells, T cells, basophils, GM-CSF.	Increased expression in macrophages infected with CAEV.	[16,52]
Interleukin 10/IL-10	DQ837159.1/Q8HY37	Immune response, immunosuppressive effect, affects monocyte/macrophage functions, inhibits expressions of IL-1α, IL-1β, IL-6, IL-10, IL-12, IL-18, GM-CSF, G-CSF, TNF-α, MCP-1α, MCP-5, MIP-1α, MIP1-β, RANTES, IL-8, chemokine receptors.	Increased expression (2 to 11 fold) in goats with the highest proviral and viral RNA loads, increased expression in the lungs of infected sheep.	[3,16,51,64]
Interleukin 12p35 subunit alpha precursor/IL-12p35 (IL-12A)	AF003542.1/O02814	Acts as a growth factor for activated T and NK cells. Enhances the lytic activity of NK/lymphokine-activated killer cells. Stimulates the production of IFN-gamma by resting PBMC.	-	[64]
Interleukin 12p40 subunit beta precursor/IL-12p40 (IL-12B)	AF007576.1/P68221	Acts as a growth factor for activated T and NK cells. Enhances the lytic activity of NK/lymphokine-activated killer cells. Stimulates the production of IFN-gamma by resting PBMC. Associates with IL23A to form the IL-23 interleukin (acting with IL-17 in an acute response to infection in peripheral tissues). Activates the JAK-STAT signalling cascade (by binding to a heterodimeric receptor complex composed of IL12RB1 and IL23R). Possibly liable for autoimmune inflammatory diseases and tumor genesis.	Increased expression (2 to 11 fold) in goats with the highest proviral and viral RNA load.	[3,64]
Interleukin 16/IL-16	AF481158.1/D2SZG5	Immune response, inhibits proliferation of T-cells, activates the release of TNF-α, IL1-β, and IL-15, inhibits IL-4 and IL-5 expression.	Increased expression (mRNA and protein level) in CAEV-infected goat blood and synovial membranes. May inhibit viral integration	[3,16,54,55,56,64]
Interleukin 17A/IL-17A	-/D2XZ62	Inflammatory response, upregulates the expression of pro-inflammatory cytokines, chemokines, and metalloproteases.	-	[64]
Interleukin 18/IL-18	AY605263.1/Q3ZT29	Interferon-gamma-inducing factor, Increases natural killer cell activity in spleen cells that stimulate interferon gamma production in T-helper type I cells.	-	[64]
Interferon alpha/IFN-α	FJ959074.1/C5IU72	Defense response to virus.	Increased expression (2 to 11 fold) in goats with the highest proviral and viral RNA loads	[3,64]
Interferon beta/IFN-β	-/K4JGW6	Antiviral defence.	Similar level of transcript and protein level in the blood on infected and healthy goats	[64]
Interferon gamma/IFN-γ	AY304501.1/P79154	Produced by lymphocytes. Antiviral activity. Important immunoregulatory functions. Activates macrophages. Anti-proliferative effects on transformed cells. Increases the antiviral and antitumor effects of type I interferons. Increases CAEV LTR activity.	Elevated expression in the lungs of infected sheep, promotes virus replication in goats	[51,65]
Tumour necrosis factor alpha/TNF-α	X14828.1/P13296	Mainly secreted by macrophages. Binds to the major receptors for tumor necrosis factor (TNFRSF1A/TNFR1 and TNFRSF1B/TNFBR). Possibly induces cell death of certain tumor cell lines. Causes fever by direct action or by stimulating interleukin-1 secretion. Induces cachexia. Possibly stimulates cell proliferation and induction of cell differentiation. Increases CAEV LTR activity	Elevated expression in the synovial membranes of infected goats and infected sheep microglia; promotes virus replication in goat, stimulates the monocytes in early response to infection	[51,52,57,61]
Monocyte chemoattractant protein-1/MCP-1/CCL2	XM_005693218.2/XP_005693275.1	The key chemokines that regulate migration and infiltration of monocytes/macrophages	Elevated expression in infected culture of macrophages	[62]
Granulocyte-Macrophage Colony Stimulating Factor/GM-CSF	DQ010419.1/AAY16326.1	Controls the production, differentiation, and function of granulocytes and macrophages	Promotes virus replication	[57]
